# Rodent models in placental research. Implications for fetal origins of adult disease

**DOI:** 10.1590/1984-3143-AR2021-0134

**Published:** 2022-04-22

**Authors:** Nicole Aguilera, Francisca Salas-Pérez, Macarena Ortíz, Daniela Álvarez, Bárbara Echiburú, Manuel Maliqueo

**Affiliations:** 1 Laboratorio de Endocrinología y Metabolismo, Facultad de Medicina Occidente, Universidad de Chile, Santiago, Chile; 2 Instituto de Ciencias de la Salud, Universidad de O'Higgins, Rancagua, Chile

**Keywords:** rodent models, maternal nutrition, hypoxia, and hyperandrogenism

## Abstract

Rodent models in rats, mice, and guinea pigs have been extremely helpful to gain insight into pregnancy physiology and pathologies-related. Moreover, they have allowed understanding the mechanism that links an adverse intrauterine environment with the origin of adult disease. In this regard, the effects of diverse maternal conditions, such as undernutrition, obesity, hypoxia, and hyperandrogenism on placental function and its long-term consequences for the offspring, have been widely analyzed through rodents models involving dietary manipulations, modifications in environmental oxygen, surgical and pharmacological procedures that reduce uteroplacental blood flow and administrations of exogenous testosterone and dihydrotestosterone (DHT) mimicking maternal androgen excess. Both in human and in rodent models, these interventions induce modifications of placental morphology, transport of glucose, amino acid, and fatty acids, steroid synthesis, and signaling pathways control placental function. These changes are associated with the increase of pro-inflammatory and oxidative stress markers. For its part, offspring exhibit alterations in organs involved in metabolic control such as the hypothalamus, adipose tissue, liver, skeletal muscle, and pancreas altering the intake and preferences for certain foods, the metabolism of glucose and lipid, and hormonal function leading to fat accumulation, insulin resistance, fatty liver, dyslipidemia, and elevated glucose levels. Therefore, the present review discusses the evidence emerging from rodent models that relate maternal nutrition, hypoxia, and androgen exposure to the maternal mechanisms that lead to fetal programming and their metabolic consequences in postnatal life.

## Introduction

Fetal programming or the developmental origin of the health and disease (DOHaD) hypothesis postulate that adverse intrauterine environment induced by deleterious environments or the presence of pathologies during pregnancy trigger fetal adaptations and, in many cases, fetal growth restriction (FGR) and increased risk of developing chronic diseases as early as at adolescence or long-term during adulthood or aging (Perrone et al., 2016). Among the factors that account for these phenomena are unhealthy maternal habits such as obesogenic diets, smoking, physical inactivity, and psychosocial stress. Complications related to pre-existing health problems or those that appear during pregnancy, like endocrine and metabolic diseases, also induce fetal programming (Perrone et al., 2016; Marciniak et al., 2017). Interestingly, maternal obesity, polycystic ovary syndrome (PCOS), gestational diabetes, and preeclampsia are related to abnormal steroid synthesis that increases androgen levels affecting fetal growth and risk of metabolic, reproductive, and psychiatric diseases.

The placenta is the main organ of pregnancy that provides the growing fetus with nutrients, participates in gas exchanges, syntheses almost all peptides and steroid hormones, and removes fetal waste products. A substantial amount of evidence indicates the primary role of the placenta in the mechanism account of fetal programming. In this regard, the changes in the intrauterine environment influence oxygen and nutrient maternal-fetal transfer through alterations of metabolic and inflammatory pathways, modifications in steroid and cytokines synthesis, and lipid and protein oxidation (Sferruzzi-Perri and Camm, 2016). Likewise, face to this environment, the fetus produces a redistribution of fetal blood flow to organs with higher metabolic demands like the heart and brain at the expense of others generating structural changes and regulating gene expression through epigenetic modifications of cell pathways that control the physiology of various organs causing fetal programming (Marciniak et al., 2017).

Animal models provide invaluable information for elucidating the mechanisms involved in fetal programming because they allow testing controlled the maternal exposure to diverse conditions that include nutritional and oxygen modifications or surgical and pharmacological paradigms that mimic multiple pregnancy pathologies (Warner and Ozanne, 2010). Because of their short gestation and lifespan, rodents are helpful and standard models for pregnancy physiology and the consequences for the offspring even more than one generation. Moreover, it is possible to obtain tissue samples from the mother, placenta, fetus, or offspring during any stages of the exposure allowing more insight into the short- and long-term relationships between mother and fetus in the development programming (Warner and Ozanne, 2010). In this regard, rats, mice, and guinea pigs are used widely for studies of nutritional interventions, maternal and fetal hypoxia, and prenatal androgen exposure. These rodent models share several similarities with the physiology of human pregnancy and placental function, giving essential information to understand the mechanism that affects fetal growth and development. However, they also show fundamental differences in the anatomy and physiology between them and in comparison to humans including uterine shape, the number of embryos, placental morphology, endocrine function, and metabolic requirements. Then, these differences should keep in mind when interpolating the findings in rodent models into human pregnancy.

Therefore, we will review and discuss the evidence emerging from rodent models that relate maternal nutrition, hypoxia, and androgen exposure to the maternal mechanisms that lead to fetal programming and their consequences in postnatal life.

## Differences between rodent and human pregnancy

Pregnancy in rodents has significant differences with humans; the most evident is the litter size (Table 1). In rats, it ranges from 10 to 16 pups; in mice is 6 to 8, and in guinea pigs, usually, the number of pups is 2 to 4 (Andersen et al., 2018). In addition, the length of pregnancy varies between species and strains, being the delivery, in rats and mice between gestational day (GD) 19 to 21, whereas in guinea pigs is 60 to 70 days (Andersen et al., 2018). According to the degree of maturation of bone, muscle, and nervous system at birth, species can be classified as precocial, giving birth to pups with well-developed sensory and locomotor skills systems like in humans, guinea pigs, and spiny mice. On the other hand, altricial such as the rat, mouse, or hamster, birth underdeveloped, relatively immobile, lack of hair, and closed eyes. Then, the correct selection of the rodent species is critical to early postnatal studies, mainly in the neurodevelopmental field.

**Table 1:** Main pregnancy and placental characteristics in human, mouse, rat and guinea pig.

	**Human**	**Mouse**	**Rat**	**Guinea pig**
	***Homo sapiens***	***Mus musculus***	***Rattus norvegicus***	***Cavia porcellus***
***Pregnancy characteristics***				
Pre-pregnancy weight (g)	6000	20	280	700
Gestation length (days)	266-280	19-22	19-22	60-70
Number of fetuses	1	6-8	10-16	2-4
Neonate weight (g)	3200	1	6	80
Neonatal maturity	Precocial	Altricial	Altricial	Precocial
***Placental characteristics***				
Placental shape	Discoid	Discoid	Discoid	Discoid
Placental barrier	Hemomonochorial	Hemotrichorial	Hemotrichorial	Hemomonochorial
Fetal-maternal interdigitation	Villous	Labyrinthine	Labyrinthine	Labyrinthine
Ovarian steroidogenesis	Progesterone until 8 wk	Progesterone	Progesterone	Progesterone until mid-pregnancy
		Estrogen	Estrogen	Estrogen
Placental steroidogenesis	Progesterone	Androstenedione	Androstenedione	Progesterone
	Estrogen			

Placenta has a maternal portion, the decidua, and a fetal part, the chorion, and it consists of mesenchymal, immune, vascular, and trophoblast cells (cyto-and syncytiotrophoblast). Cytotrophoblasts fuse to form the syncytiotrophoblast, a specialized epithelium, carries out gas exchange and expresses nutrient transporters for glucose, amino acid, and fatty acid. Among the species, there are differences in placental shape, degree of the relationship between the chorion and uterine wall, number of layers of trophoblast, and maternal-fetal interdigitation (villous, trabecular, or labyrinthine)

**Figure 1:**
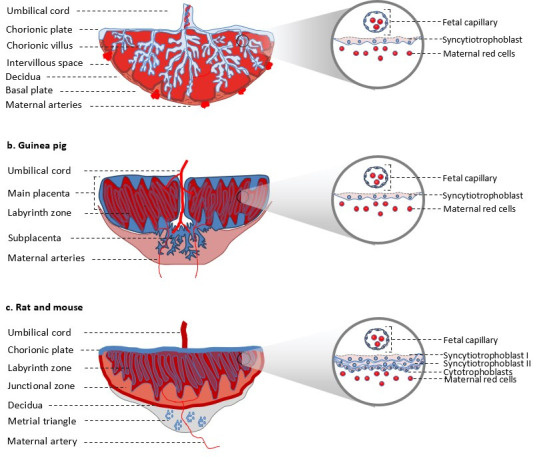
Schematic representation of placenta from human (a); guinea pig (b); and mouse or rat (c). The inset shows a magnification of the placental barrier.

According to the relationship between the chorion and uterine wall, the placenta is classified as epitheliochorial, in which fetal tissue is closely in contact with maternal uterine epithelium but does not invade it significantly. This type is found in horses, pigs, and ruminants. In contrast, those in which the trophoblast invades in different degrees the uterine wall are: the endotheliochorial that is present in eutherian mammals and carnivores, and it is characterized by a trophoblast invasion up to the level of the basal lamina of maternal endothelial cells. Humans and rodents possess a hemochorial placenta, in which cytotrophoblasts invade and replace endothelial and smooth muscle cells in spiral arteries remodeling from high-resistance/low-capacity to low-resistance/high-capacity vessels facilitating uterine blood flow and placental perfusion (Furukawa et al., 2014) (Figure 1). In guinea pigs and humans, there is only one layer of trophoblasts that separates the maternal blood space from the fetal capillaries; then, it is also classified as hemomonochorial (Figure1a and b). In contrast, in mice and rats, the placenta is hemotrichorial because there are three layers, one of cytotrophoblast and two of syncytiotrophoblasts, between maternal blood space and fetal capillaries (Figure 1c) (Andersen et al., 2018).

The branching of the chorion forms the chorionic villi, which is the functional unit because it participates in maternal/ fetal exchange and hormone synthesis. Chorionic villi interdigitate into the intervillous space that receives maternal blood via the spiral arteries (Figure 1a). The placenta of guinea pigs is composed of labyrinthine lobes, where syncytiotrophoblast is embedded in maternal blood. The subplacenta connects the main placenta with the junctional zone and serves as the source of extravillous trophoblasts that invade the endometrium and have endocrine functions (Figure 1b) (Capellini et al., 2011). In rats and mice, the placenta contains a fetal part that includes the labyrinthine zone, which meets the same exchange function of intervillous space in humans, the junctional zone that participates in hormone synthesis, and the yolk sac, which is not present in humans. The maternal part is formed of the decidua and metrial gland (Figure 1c).

Sex steroids play a central role in pregnancy, regulating immune tolerance, maternal food intake, glucose and lipid metabolism, uterine blood flow, placental angiogenesis and nutrient transport, and the timing of labor. In this regard, steroid receptors, including progesterone, estrogen, glucocorticoids, and androgen, are present in the placenta from mice, rats, and guinea pigs, indicating that steroid action regulates rodent placenta. However, there are more differences than similarities regarding steroid synthesis between humans and rodents (Table 1).

In humans, progesterone is produced at the beginning of pregnancy by corpora lutea under the control of chorionic gonadotropin secreted by the syncytiotrophoblast. From about eight weeks of gestation, progesterone is synthesized exclusively in the placental trophoblast. However, progesterone supporting pregnancy is produced mainly in mice and rats' ovaries (Warshaw et al., 1986; Strauss et al., 1996). On the other hand, guinea pigs, like humans, exhibit a luteo-placental shift in hormone production (Csapo et al., 1981). For its part, estrogens are produced from androgen precursors aromatized by P450 aromatase in the human placenta. On the contrary, the mouse and rat placenta expresses 17-hydroxylase activities, which is critical to synthesize androgens from progestogens, then can produce *de novo* androgens that are utilized as substrate by ovarian P450 aromatase to synthesize estrogens. Unlike humans, the placenta does not express aromatase activity in these species.

## Rodent models of dietary interventions, hypoxia, and hyperandrogenism.

**Figure 2:**
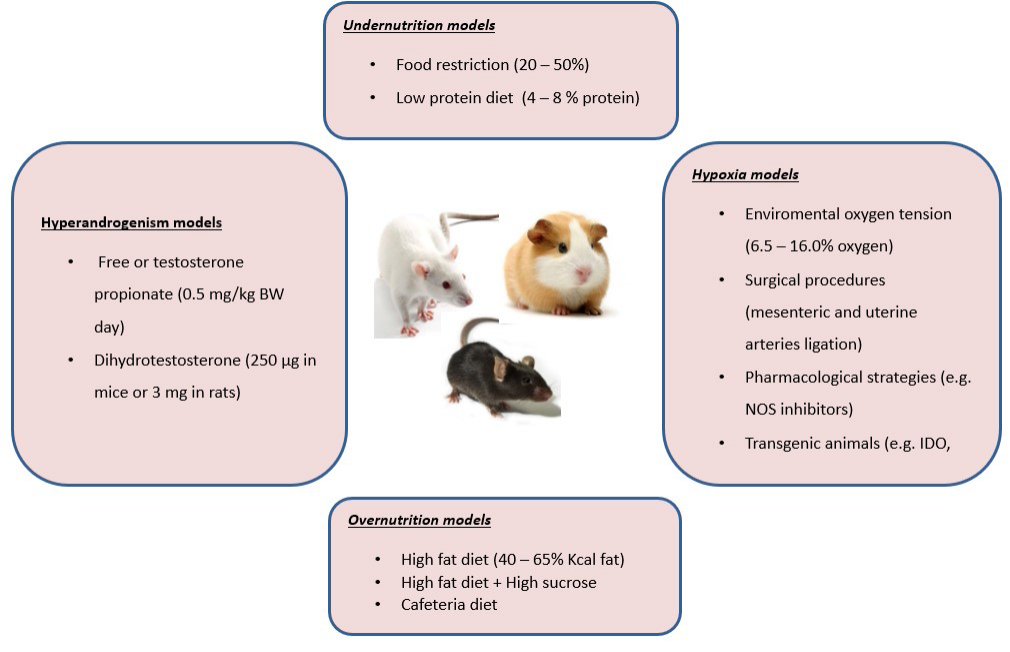
Rodent models of dietary interventions, hypoxia, and hyperandrogenism.

The interventions in rodent models that allow evaluating the effects of nutrition comprise under and over-nutrition with modifications in the energy content and nutrients components. In turn, hypoxia models can be reached through surgical techniques, environmental modifications, and genetic manipulations. On the other hand, gestational hyperandrogenism can be induced pharmacologically with hormones with androgens activity, mainly testosterone and dihydrotestosterone. These interventions are summarized in Figure 2 and will be described in the following sections.

Rodent models of caloric and nutrient manipulations have provided relevant insights into the effects of maternal undernutrition and obesity on placental nutrient transport, diet-associated mechanisms of fetal growth, and its long-term consequences for the offspring. The most extensively dietary rodent models of intrauterine growth restriction (IUGR) have applied a reduction of 20 to 50% of food intake or low protein diet (LPD) ranging from 4 to 8% of total calories instead of 19 to 22% in control chow (Warshaw et al., 1986). On the other hand, to test nutrient excess, rodent models have involved the exposure, from before and during gestation, to high-fat (HFD) containing 18 to 65% of calories as fat with or without elevated levels of carbohydrates supplied as sucrose into the pellet diet or in drinking water (Williams et al., 2014). Another strategy that resembles human dietary habits is the cafeteria diet that includes human snack food items, i.e., cheesecake, chocolates, fried chips, and cookies, into the standard chow (Neri and Edlow, 2015).

Oxygen is critical for mammals because it is a substrate for oxidative metabolism, and oxygen deprivation can damage several tissues. In physiological conditions, oxygen levels are nearly 20 mL O_2_/dL with slight differences in women due to minor hemoglobin levels, which mediates oxygen transport (Olive et al., 2018). Prenatal hypoxia can originate from maternal, placental, and fetal conditions. Kingdom and Kaufmann classified hypoxia as pre-placental if the mother and fetus are affected due to external hypoxic environments (i.e., living at high altitudes, pulmonary hypertension, maternal anemia); uteroplacental due to an abnormal blood flow to these tissue product of occlusions or failed trophoblast invasion; and post-placental when the fetus is hypoxic due to fetal diseases (Kingdom and Kaufmann, 1997). It has been postulated that an abnormal trophoblast invasion leads to placental ischemia promotes chronic inflammation, endothelial dysfunction, and increased oxidative stress (Khalil and Granger, 2002; Redman, 2011). Guinea pigs have been described as an excellent animal model to study the effects of hypoxia on placentation mechanisms because they present deep trophoblast invasion into the maternal decidua and a more extended gestational period than rats or mice species (Morrison et al., 2018).

An abnormal uteroplacental blood flow has been implicated in preeclampsia's pathogenesis, which is pregnancy-related, defined as new-onset hypertension and proteinuria after 20 weeks of gestation. Preeclampsia is strongly associated with FGR and maternal morbidity because it can cause eclampsia, a severe and high-mortality pregnancy-pathology.

Preeclampsia can evolve in two stages: early-onset preeclampsia due to a defective placenta formation or a reduced uteroplacental blood flow, and late-onset preeclampsia, associated with placental pathologies (Roberts and Hubel, 2009). Animal models for the study of preeclampsia can be divided into four categories according to their mechanisms, as previously reported: spontaneous animal models of preeclampsia, surgical interventions, pharmacologically/substance-induced models, and transgenic animal (Erlandsson et al., 2016).

The BPH/5 mouse mimics both early and late-onset preeclampsia, showing elevated blood pressure, endothelial dysfunction, glomerular lesions, proteinuria, and fetoplacental defects (Davisson et al., 2002). On the other hand, surgical models involve mechanical occlusions in the uterine artery or the abdominal aorta that reduce uteroplacental perfusion (Li et al., 2012). Pharmacologically induced models have shown that exposure to l-NAME, an inhibitor of nitric oxide synthase (NOS), or arginine vasopressin (AVP) can replicate classic maternal and fetal preeclampsia symptoms (Molnár et al., 1994; Santillan et al., 2014). Finally, several genetically modified animal models simulate preeclampsia, including knockout (KO) mice for indoleamine 2,3-dioxygenase (IDO) that regulates endothelial-derived relaxing factors (Santillan et al., 2015). Moreover, pregnant interleukin-4 (IL-4 -/-) exhibit mild preeclampsia-like symptoms, and interleukin-10 (IL-10 -/-) knockout mice exposed to hypoxia present placental injury, proteinuria, and hypertension.

In humans, increased circulating testosterone levels are consistently found in maternal malnutrition (Maliqueo et al., 2017; Barrett et al., 2019), gestational diabetes (Villarroel et al., 2017), preeclampsia (Troisi et al., 2003), and polycystic ovary syndrome (PCOS) (Maliqueo et al., 2015; Caanen et al., 2016). This latter is the most common endocrine-metabolic condition in women characterized by hyperandrogenism and oligo-anovulation, leading to reduced fertility and metabolic disorders like obesity, dyslipidemia, and type 2 diabetes (Boyle and Teede, 2016). It is noteworthy that elevated maternal androgens are associated with placental dysfunction and FGR (Carlsen et al., 2006; Kumar et al., 2018). Rodent models in rats and mice involving subcutaneous injections of testosterone or dihydrotestosterone (DHT) at the last third of pregnancy have been commonly used to understand the role of androgens on placental function, fetal growth, and long-term consequences for offspring. Models in rats comprise the administration during five consecutive days of 0.5 mg/kg/day of testosterone (GD15 - GD19) or 3.0 mg/day of DHT during four days (GD16 - GD19) (Sathishkumar et al., 2011; Dean et al., 2012; Sun et al., 2012). For its part, mice models have used subcutaneous administration of 250 μg of DHT from GD16.5 - 18.5 (Fornes et al., 2017). Moreover, this latter has been combined with the administration of high fat and high sucrose diet to understand the relationship between endocrine abnormalities and metabolic disorders (Risal et al., 2019).

## Placental adaptations in rodent models of dietary interventions, hypoxia, and hyperandrogenism

Food and chronic protein restrictions in rats and mice reduced maternal weight gain at the last third of pregnancy; and the placental and fetal weights (Jansson et al., 2006; Gonzalez et al., 2016; Connor et al., 2020). Similarly, in C57BL/6J mice, it has been reported that four days of exposure to hypoxia (10.5% O2) at mid-pregnancy caused a decrease in the number of viable pups and a reduction of 36% in birth weight at gestational day 18.5 without changes in placental weight (Cuffe et al., 2014). In rat models, it has been reported that three days of hypoxia (12% O2) from GD18 until GD21 can induce a reduction of 10% in birth weight without changes in litter size (Thaete et al., 2004). Similarly, hypoxia levels for a longer period, starting from GD14 until GD20, can induce a reduction of 20% in weight (Jakoubek et al., 2008). Even more, hypoxia for 11 days causes a severe FGR with a decrease of 30% fetal-weight with smaller litter size and lower placental weight (Huang et al., 2004). In guinea pigs, hypoxia-induced by 10.5% O2 reduced fetal weight but increased placental weight (Thompson et al., 2016). Meanwhile, in rats, maternal exposure to testosterone or DHT and in mice to DHT produce lower fetal weight and reduced placental size (Sun et al., 2012; Risal et al., 2019).

The effects on fetal growth observed by nutritional interventions, hypoxia, and hyperandrogenism are likely related to placental modifications due to reduced uteroplacental blood flow and nutrient transport. Thus, it is common to observe smaller placentas with alterations in specific placental zones that account for the modifications in nutrient transport and hormone synthesis. Both under and overnutrition-induced diets mainly affect the placental junctional zone, reducing the number of glycogen trophoblast cells, which meets a nutritional function. Moreover, rats fed with LPD increased density and number of spongiotrophoblast and giant cells producing prolactin-like, and lactogens hormones that maintain progesterone secretion from the corporea lutea and cytokines that regulate placental functions in the junctional zone were observed (Vomhof-DeKrey et al., 2016). These data indicate that maternal undernutrition launches nutritional and endocrine compensatory mechanisms to pregnancy maintenance and avoid growth restriction; however, these seem to be insufficient considering that reduced fetal growth. LPD in rats could also affect steroid synthesis because it decreases the gene expression of 17-beta-hydroxysteroid dehydrogenase type 2 in the junctional zone in both female and male fetuses (Gao et al., 2012).

In guinea pigs, prenatal hypoxia (10.5% O_2_) during early gestation inhibits cytotrophoblast invasion of spiral arteries that affects placentation increasing maternal blood pressure that resembles changes observed in women with preeclampsia (Turan et al., 2017). On the other hand, it was observed that hypoxia also induced a compensatory blood vessel expansion in the guinea pig labyrinth in response to inhibited upstream arterial remodeling (Thompson et al., 2016). Interestingly, in CD1 mice, placentas of female fetuses responded differently to maternal hypoxia, showing reduced placental labyrinth blood spaces, which was not observed in male placenta (Cuffe et al., 2014). Overall, maternal exposure to hypoxia or surgical restriction of uterine blood flow induce placental hypoxia, evidenced by hypoxyprobe staining or protein expression of hypoxia-inducible factor 1α (HIF1α), increased apoptosis, reactive oxygen species (ROS), and mitochondrial or endoplasmic reticulum stress (Siragher and Sferruzzi-Perri, 2021). Placental transcriptome analysis in mice exposed to 10.5% oxygen from GD 14.5 to 18.5 showed an altered expression of genes involved in vasculature development, hemostasis, adhesion, and extracellular matrix despite no changes in placental and fetal weights (Chu et al., 2019).

In this same line, maternal LPD decreased M1 macrophages and increased M2, producing TNFα, and invariant natural killer T (iNKT) cells, secreting significant amounts of cytokines in response to glycolipids, also suggesting a pro-inflammatory state. Similarly, HFD (45% kcal as fat) in pregnant mice significantly decreased placenta labyrinth thickness, where maternal-fetal exchange occurs, showing lower cell proliferation, increased macrophage activation, and elevated pro-inflammatory cytokine gene expression (Kim et al., 2014).

Along with the changes described above, LPD overexpressed placental angiogenic factors such as fibroblast growth factor 2 (FGF2), vascular endothelial growth factor receptor 1 (VEGFR1), and insulin-like growth factor 2 (IGF2) contributes to placental vascular defects because did not revert the FGR (Vomhof-DeKrey et al., 2016). Similarly, maternal undernutrition downregulates VEGF and several antioxidant enzymatic systems that could compromise oxygen delivery and increase oxidative stress, mainly in the male fetus and moderately in females (Phuthong et al., 2020). On the other hand, hypoxia models increase placental expression of angiogenesis factors, including Flt1 and VEGF (Thompson et al., 2016; Natale et al., 2018). In the same way, mice fed with a highly palatable obesogenic diet supplemented with sweetened condensed produced an impaired labyrinth development associated with dysregulation of transcripts and pathway interactions related to placental vasculature and structure (Barros Mucci et al., 2020). Interestingly, HFD also induced labyrinth placental endothelial damage in mice, which seems to be associated with oxidative stress because treatment with quercetin, an antioxidant, reverted this effect (Liang et al., 2010).

Pregnancy interventions have important implications in the pathways regulating placental nutrient transport, causing maternal-to-fetal nutrient transfer modifications. In this regard, rats fed with an isocaloric diet containing 4% of protein resulted in placental down-regulation of the system A and L amino acid transporters that uptake alanine, serine, proline, glycine, tryptophan, and neutral and branched-chain amino acids. However, glucose transport seems not to be affected despite the changes in gene expression of facilitated glucose transporter member 1 (*Slc2a1*) (Coan et al., 2010). Regarding the placental fatty acid transporters, in food-restricted mice, this was dependent on fetal sex, showing that protein expression of fatty acid transporter protein (FATP) 4 and gene expression of the fatty acid-binding protein plasma membrane (*fabppm)* were higher, and fatty acid translocase (*Fat/Cd36*) lower in male placentas. In contrast, endothelial lipase (*El*) was higher in females than in male placentas (Connor et al., 2020).

Similarly, Sprague-Dawley rats fed with 40% calories as fat show placental lipid accumulation associated with lower mRNA levels of crucial lipid transport and storage genes (*Cd36, Fabp3, Pparg, Plin2, and Irs1*) (Louwagie et al., 2018). These changes were concomitant with the inhibition of placental signaling for key metabolic pathways, including insulin, mammalian target of rapamycin (mTOR), and signal transducer and activator of transcription 3 (STAT3) (Rosario et al., 2011). On the other hand, maternal obesity-induced by HFD (41 kcal% fat) supplemented with 20% of sucrose showed increased activity and protein expression of the system A and L amino acid transport in the placental barrier, in addition to higher expression of glucose transporters, which was associated with fetal overgrowth (Rosario et al., 2015). Interestingly, signaling pathways are associated with energy metabolism, such as mTOR, insulin, growth factors, and leptin was overactivated, explaining the stimulation of placental nutrient transporters (Rosario et al., 2016).

Meanwhile, 13% oxygen during GD14 to GD19 in C57BL/6 mice increased placental glucose transfer without affecting the amino acid transfer. On the other hand, 10% oxygen reduced amino acid transport without modifications in glucose transport (Cuffe et al., 2014). In addition, in placentas from CD1 mice exposed to hypoxia, mRNA expression of *Glut1, Igf2 and Igf1r* was reduced only in female placentas (Cuffe et al., 2014). These changes were associated with alterations in the insulin-IGF signaling pathway in the labyrinth zone. Interestingly, the lowest oxygen levels produced a higher FGR (Higgins et al., 2016). Moreover, increased Akt-mTOR signaling in the hypoxic placentas consistent with heavier placenta was also observed (Matheson et al., 2016). In addition, another study that analyzed placental transcriptome analysis confirms these observations, including also, MAPK and genes associated with inflammatory responses (Chu et al., 2019). In the same way, AMPK was activated in the uterine artery and the labyrinth zone in response to 10% hypoxia (Skeffington et al., 2016)

Testosterone during gestation leads to reduced placental system A amino acid transport activity, which seems to be associated with the downregulating of amino acid transporter *slc38a2/Snat2*. On the other hand, testosterone exposure did not modify glucose transport capacity (Sathishkumar et al., 2011). Although alterations in nutrient transport were not related to mTOR signaling, as in the case of dietary modifications or hypoxia, overactivation of the STAT3 pathway was observed (Hu et al., 2015). Interestingly, testosterone exposure also leads to alterations in the expression of estrogen and androgen receptors and 17-beta-hydroxysteroid dehydrogenase type 2 (17β-HSD2) that suggest an altered placental steroid signaling and steroidogenesis (Sun et al., 2012). However, of these models is not possible to dissect the role of estrogen because testosterone, under the action of P450 aromatase, is aromatized to estradiol. To address this dilemma, non-aromatizable androgens such as dihydrotestosterone (DHT) or blocking androgen or estrogen action like flutamide or tamoxifen, respectively, have been used (Walters, 2016). In this regard, it has been observed that in mice, the administration of 250 µg of DHT at late pregnancy reduced placental and fetal weight affecting androgen and estrogen receptor expression similarly to testosterone administration (Fornes et al., 2017; Risal et al., 2019).

## Fetal programming in rodent models of dietary interventions, hypoxia, and hyperandrogenism

The placental changes originated from maternal dietary interventions, hypoxia, and elevated androgen levels lead to adaptations that redistribute fetal blood flow to ensure oxygen and nutrient supplies to organs with higher metabolic demands like the heart and brain at the expense of others. However, these changes can induce modifications and damage in specific organs that generate, among others, metabolic and cardiovascular disorders (Peeters et al., 1979; Fajersztajn and Veras, 2017).

Rodent models of maternal undernutrition have demonstrated that offspring showing reduced pancreatic β cells, insulin resistance, alterations in the regulatory mechanisms that favor energy storage after birth, despite adequate nutrients availability, resulting in obesity, diabetes, and various metabolic disorders (Hales and Barker, 2001; Marciniak et al., 2017). Obesity in offspring born to food-restricted mothers results from hyperphagia and adipocyte dysfunction. Maternal undernutrition induces higher hypothalamic expression of the orexigenic peptides agouti-related protein (AgRP) and neuropeptide Y (NPY) and lowers gene expression for the anorexigenic peptide proopiomelanocortin (*Pomc*) (Vickers et al., 2000; Fukami et al., 2012). Interestingly, LPD throughout pregnancy enhances the preference for fatty foods in the offspring, which is associated with increased motivation for food reward and an altered expression pattern of opioid receptors and other reward-related genes in the nucleus accumbens and other structures of the brain (Bellinger et al., 2004; Vucetic et al., 2010; Alves et al., 2015).

Maternal LPD during gestation and lactation affects adipose tissue by reducing the adipocyte size of rat offspring. In contrast, rat offspring from food-restricted dams showed hypertrophic adipocytes. Moreover, increased catecholamine levels and adrenoreceptors and upregulation of CCAAT/Enhancer-binding protein α (*Cebpa*) and peroxisome proliferator-activated receptor-gamma (*Pparg*), regulators of fatty acid oxidation, gene expressions were observed in adipocytes, indicating an altered lipogenesis (Petry et al., 2000; Lecoutre and Breton, 2015). In the liver of growth-restricted male offspring, elevated levels of triglycerides along with increased protein expression levels of lipoprotein lipase (LPL) were explained by an increase in the hepatic expression levels of liver X receptors (LXRs)-α expression that binds to the putative response elements in the LPL promoter regions (Zhu et al., 2016). In the same way, Wistar rats born to dams fed with 50% restricted diet showed induction of cholesterol biosynthesis with higher concentrations of very-low-density lipoprotein (VLDL) and low-density lipoprotein (LDL), lipoproteins, and triglycerides (Sarli et al., 2021). Therefore, abnormalities in adipose tissue and liver lead to dyslipidemia with metabolic consequences. In the same way, hyperinsulinemia and reduced glucose uptake insulin-stimulated and expression of total and phosphorylated specific insulin-signaling proteins in skeletal muscle have also been demonstrated in male and female offspring at 15 and 21 months old, respectively, indicating an insulin-resistance state induced by maternal LPD during pregnancy (Fernandez-Twinn et al., 2005).

In rodent models of maternal overnutrition, offspring present weight gain, hyperphagia (Ozanne et al., 2004), leptin resistance (Samuelsson et al., 2008; Morris and Chen, 2009) and hyperleptinemia (McMillen et al., 2006), increased adiposity (Li et al., 2013), arterial hypertension (Samuelsson et al., 2008; Elahi et al., 2009), insulin resistance, higher hepatic gluconeogenesis (Strakovsky et al., 2011; Taylor et al., 2014), lipid profile abnormalities (Elahi et al., 2009), in addition to increasing pro-inflammatory mediators and vascular endothelial dysfunction, which are key factors in the development of metabolic syndrome, fatty liver, hepatic inflammation and steatosis (Li et al., 2013) and cardiovascular disease (Vickers et al., 2000; Ghosh et al., 2001; Khan et al., 2005; Chen et al., 2008; Ashino et al., 2012). Metabolic alterations in offspring born to mothers fed with high-fat diet consumption appear early during postnatal life. In Sprague–Dawley rats, maternal HFD induced intrauterine inflammation, showing enhanced inflammatory cytokines (IL6, IL-1β, and TNF-α) in umbilical cord blood and the placenta that contribute with metabolic disorders in neonates characterized by alterations in hepatic genes both lipid synthesis- and β-oxidation that promote hepatic lipid accumulation (Cao et al., 2020). On postnatal day 10, they exhibit, in a sex-specific manner, changes in dopamine-related gene expression (*Th* and *Slc6a3*, also known as *Dat1*) in the hypothalamus that could contribute to hyperphagia and preference for fatty, sugary, and salty foods at the expense of protein-rich foods (Bayol et al., 2007; Barrand et al., 2017). Both male and female offsprings show increased adiposity, which is more pronounced in females than males. In the same way, elevated expression of genes involved in insulin signaling (*Igf1* and *Irs1*), angiogenesis (*Vegfa*), lipogenesis (*Pparg* and *Lpl*), adipocyte function (*Lep* and *Adipoq*), and glucose uptake (*Slc2A1* and *Slc2A3*) in females fed cafeteria diet compared with females did not have access to this diet (Bayol et al., 2008).

Therefore, rodent models of maternal overnutrition in mice and rats show similar alterations in the hypothalamus, adipose tissue, muscle, and liver than those observed in models of undernutrition, although these appear earlier than offspring born to overnourished than undernourished mothers.

Like those observed in nutritional interventions, hypoxia can lead to cardiovascular and metabolic alterations in the offspring. In adulthood, the hearts of offspring express abnormal phenotypes, including diastolic dysfunction, increases in myocardial contractility, and responsiveness to β-adrenoreceptor stimulation (Giussani et al., 2012; Niu et al., 2018). Although these alterations are developed over time during postnatal life because fetal heart function measured *in vivo* is not disrupted by hypoxia, despite FGR, suggesting a functional capacity to adapt to prolonged hypoxic stress (Thompson et al., 2016). Interestingly, in deer mice (*Peromyscus maniculatus*), a native rodent to high altitude (4350 m.a.s.l), hypoxia exposure during early life likely contributes to the ability to cope with hypoxia, increasing their plasticity on metabolism, oxygen consumption rate, and body temperature regulation (Ivy and Scott, 2021). Likewise, FGR induced by bilateral uterine ligation alters nephrogenesis, leading to increased serum corticosterone levels, decreased nephron number, and cause adult-onset hypertension (Baserga et al., 2007).

Rodent models of hypoxia exposure during gestation show gene and protein expression changes in different organs involved in nutrient metabolism. Maternal hypoxia increased body weight and food consumption, reduced daily energy expenditure, increased adiposity index and insulin resistance, systemic elevations of lipid levels, and altered macrophage populations in adult male, but not female, mice offspring (Khalyfa et al., 2017). Interestingly, these effects can be attributed to altered leptin action in hypothalamic arcuate nuclei in male offspring in the Sprague-Dawley rat (Vargas et al., 2017). Moreover, reduced protein phosphorylation related to insulin signaling and lipid accumulation in the liver, along with lower expression of GLUT4 in skeletal muscle, were also observed (Camm et al., 2011; Cao et al., 2012). It is interesting to note that gestational hypoxia leads to abnormalities in maternal lipid and carbohydrate metabolism, suggesting that described effects in the offspring can result from alterations in maternal-fetal nutrient transport (Määttä et al., 2018).

In females, exposure to chronic maternal hypoxia during fetal development leads to accelerated ovarian aging of somatic cells and reduced ovarian reserve at pubertal age, associated with gene pathways regulating folliculogenesis and steroidogenesis (Aiken et al., 2019; Pampanini et al., 2019). In turn, male offspring exhibited abnormalities in the proliferation and differentiation of spermatic cells and alterations in angiogenesis and connective tissue growth. Thus, together with metabolic and cardiovascular alterations, maternal hypoxia also results in reproductive dysfunctions.

Differences in the timing of exposure to androgen excess lead to variations in the appearance of PCOS features observed in animal models, implying that developmental stages are likely to be a key determinant in PCOS pathogenesis (Caldwell et al., 2014). Rats and mouse models of prenatal exposure to testosterone or DHT excess have been reported to induce most of the relevant reproductive, endocrine, and metabolic features of PCOS (Walters, 2015). Longer anogenital distance and smaller weight are frequent features observed in female pups born to androgenized rats and mice (Sun et al., 2012; Risal et al., 2019). Moreover, they had irregular cycles, polycystic ovary morphology, lower androgen levels being DHT lower in rat models, and testosterone and androstenedione in mice models (Hu et al., 2015). Along with reproductive phenotype, metabolic and cardiovascular abnormalities have been described, including clear signs of non-alcoholic fatty liver (Sun et al., 2012), which seems to be established during fetal life such that the expression of the transcription factor *Pparg* was decreased in fetal livers exposed to DHT (Fornes et al., 2017), in addition, insulin resistance, alterations in the adipocyte functions and adipogenesis, and dyslipidemia have also been described (Sun et al., 2012; Risal et al., 2019). Elevated blood pressure has been demonstrated in rat models showing different regulatory mechanisms being NO-related in females and endothelium-derived hyperpolarizing factor (EDHF) -related in males. Sex steroids, in general, are central in the regulation of neurodevelopment processes; therefore, it is expected that both testosterone and DHT affect the behavior, in this regard, anxiety-like behavior in females, which is associated with decreased gene expression of androgen receptor (*Ar*) and increased expression of GABAergic and serotoninergic receptors, and genes involved in calcium signaling, among others, causing an anxiety-like behavior increased anxiety-like behavior (Hu et al., 2015; Risal et al., 2021). Interestingly, these alterations can be transgenerationally transmitted by maternal and paternal lineage according to DHT-mice modes have demonstrated (Risal et al., 2019, 2021)

In rodents, early postnatal exposure to dihydrotestosterone (DHT) produces the closest PCOS-like phenotype because the differentiation of reproductive tissues occurs during neonatal life contrary to humans (Caldwell et al., 2014; Kauffman et al., 2015; Walters et al., 2018). The perinatal androgenization induces irregular cycles, oligo-anovulation, PCO morphology, increased preantral/antral follicles, hyperandrogenism, LH hypersecretion, and disturbances in fat metabolism without insulin resistance. One study that evaluated pre and postnatal androgen-induced-PCOS mouse models found that a model using DHT treatment in early postnatal provided the highest concordance with clinical features of PCOS (Caldwell et al., 2014). In summary, analysis of androgenized rodent PCOS animal models has demonstrated closely mimic human PCOS as androgen excess consistently induces a wide breadth of characteristics of these disorders, which may vary depending on the type of androgen used and the timing of the androgenization.

## Conclusions

**Figure 3:**
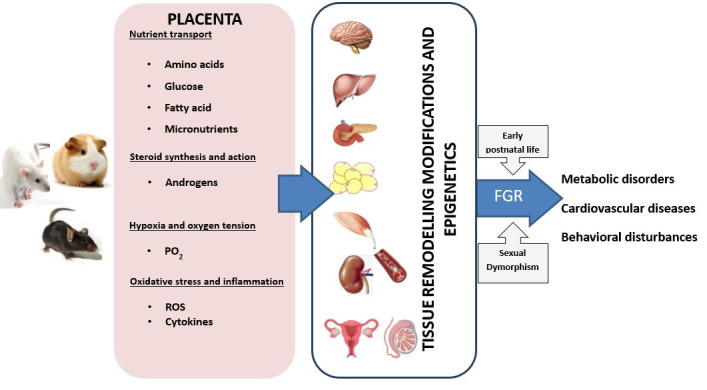
Schematic representation of the effects of dietary interventions, hypoxia, and hyperandrogenism based on observations obtained by rodent models in rats, mice, and guinea pigs. These models have shown that the mechanisms of fetal programming are mediated by placental dysfunctions involving nutrient transport, steroid action (androgens), reduced oxygen supply (modifications in uteroplacental blood flow), increased in pro-inflammatory cytokines and reactive oxygen species (ROS), leading mainly to fetal growth restriction (FGR). These modifications in the prenatal environment affect various fetal organs resulting in metabolic, cardiovascular, and behavioral disorders in postnatal life mediated by epigenetics modifications, sex dimorphism, and early postnatal life.

Rodent models of dietary interventions, hypoxia, and hyperandrogenism have shown that the mechanisms of fetal programming are mediated by placental dysfunctions (Figure 3). These alterations in insulin, mammalian target of rapamycin (mTOR), and signal transducer and activator of transcription 3 (STAT3) pathways are associated with modifications in uteroplacental blood flow, amino acids, and fatty acid transport, increased in pro-inflammatory cytokines and ROS lead mainly to FGR.

Modifications in the prenatal environment affect fetal organs such as the brain, adipose tissue, liver, heart, and endothelium, programming its function at postnatal life inducing hyperphagia, obesity, hepatic fat accumulation, blunted insulin signaling in muscle resulting in insulin resistance and cardiovascular disorders. In these alterations, sex dimorphism plays a central role such that females and males exhibit differential phenotypes.
